# The Practice of Vigorous Physical Activity Is Related to a Higher Educational Level and Income in Older Women

**DOI:** 10.3390/ijerph182010815

**Published:** 2021-10-14

**Authors:** Rafael Zapata-Lamana, Felipe Poblete-Valderrama, Igor Cigarroa, María Antonia Parra-Rizo

**Affiliations:** 1 Escuela de Educación, Universidad de Concepción, Los Ángeles 4440000, Chile; rafaelzapata@udec.cl; 2 Departamento de Ciencias del Deporte y Acondicionamiento Físico, Facultad de Educación, Universidad Católica de la Santísima Concepción, Concepción 4030000, Chile; felipepobleteva@santotomas.cl; 3 Escuela de Kinesiología, Facultad de Salud, Universidad Santo Tomás, Santiago 8320000, Chile; icigarroa@santotomas.cl; 4 Department of Health Psychology, Faculty of Social and Health Sciences, Campus of Elche, Miguel Hernandez University (UMH), 03202 Elche, Spain

**Keywords:** aging, quality of life, women, physical activity, economic resources

## Abstract

Few studies have shown evidence about the factors that can determine physical practice in women over 60 years of age due to educational, economic, social, or health inequalities. Its knowledge could help to understand the determinants that encourage the practice of physical activity and the improvement of health in women over 60. Therefore, the aim of this research was to evaluate the level of studies, income, and the usefulness of social and health services in physically active older women according to the level of activity they practice. The IPAQ (International Physical Activity Questionnaire) and CUBRECAVI (subjective health scale) scales have been applied to a sample of 257 women between 61 and 93 years old (M = 69.44, SD = 4.61). The results have shown that those with vigorous physical activity are related to higher levels of education (*p* < 0.001) and income (*p* = 0.004). Furthermore, being dissatisfied with social and health services is associated with low levels of physical activity (*p* = 0.005). Older women who perform physical activity regularly are associated with high levels in some of the socio-environmental aspects of quality of life. High physical activity is related to a higher educational level and income. Socio-environmental factors generate social inequalities and modulate the lifestyles of older women.

## 1. Introduction

The practice of physical activity on a regular basis provides physical and psychological benefits to the human being [1]. In addition, this same organization indicates that physical activity is a necessity with a population, multi-sector, and multi-disciplinary scope [2].

In this regard, the World Health Organization (WHO) (Decade of Healthy Aging 2021–2030), the European Union (EU) (Analytical Report of the Active Aging Index 2018), and the Government of Spain (National Strategy for the Active and Healthy Aging) have launched various initiatives that aim to minimize the negative impact that aging has on the population [3], above all because an active and healthy life is vital for an increasingly aging society [4].

However, a study [5] carried out with older people between 65 and 74 years old stated that only 6% of the sample practiced physical activity in their free time and highlighted the need to increase these levels of physical activity in different sectors of the population [6].

In this respect, one sector of the population that has received scientific attention regarding the practice of physical activity is the group constituted by the elderly [7] However, despite the fact that the effects of physical activity on cognitive impairment and other pathologies have been extensively studied [8], the profile of active older women practicing physical activity in connection with socio-environmental inequality and quality of life has not been thoroughly discussed. Thus, knowing what factors influence the practice of physical activity in women of this age can help establish programs that take these variables into account and, consequently, improve adherence [9]. Similarly, this highlights the importance of knowing what behaviors favor positive health outcomes in this age group, which would also help when improving intervention programs [10].

Furthermore, a study [11] with a sample of 9442 women aged 65 or over established that among the factors that determined a greater practice of physical activity was higher education compared to secondary school. The main activity these women carried out was walking; second, swimming and cycling; and to a lesser extent (less than a third) was the practice of medium or high intensity physical activity. Along the same lines, it also highlights that the level of studies is important: the higher the level of the former, the more the physical activity [12]. Additionally, a study has pointed out the relationship between the practice of physical activity and the years of schooling [13].

Thus, education has a positive effect on health. In a study carried out in 29 countries based on the European Social Surveys, the authors pointed out the decisive impact that education has on health [14]. In this way, the results of a study carried out with a sample of 383 older adults from southern Italy showed that educational level was positively associated with physical activity and therefore recommended its practice among older people with low educational levels and in women 16 [15].

On the other hand, various researchers have indicated that the economic precariousness of women between the ages of 25 and 65 can also predict the lack of sports [16] as well as of health care [10]. In this regard, in a meta-analysis performed with 19 studies related to the factors that limited the practice of physical activity in older adults, low income was highlighted [17], and a study carried out with a sample made up of 1501 Korean adults older than 60 years also connected the economic level with a sedentary lifestyle [18] as well as a research conducted from a random sample of older adults in Spain [19]. Then again, a research that analyzed the physical activity patterns of 140 low-income Brazilian women found that they had a participation of less than 600 MET (Metabolic Equivalent of Task)-min [20]. In addition, a study conducted with men also found a relationship between high income and higher practice of physical activity [21].

Another aspect that this research aims to address is the relationship between the practice of physical activity and social and health services. It draws attention to the need to increase the practice of physical activity because it also helps to reduce drug use, improves self-esteem, generates a lower risk of dependence [22], and mitigates depression among older people [23]. Despite these benefits, on this occasion, studies on older women practicing physical activity and the influence of social and health services have not been found.

Considering the limited literature found on the impact that certain external aspects (education, income, and social health services) have on the practice of physical activity and health in adult women practicing physical activity, and the shortage of studies that address this issue, the general goal of this research was to study whether older adult women who practiced a high level of physical activity scored higher at the levels of socio-environmental factors of quality of life compared to those who carry out moderate or low levels of physical activity. In particular, is important to understand the following aspects:

(a) If there are differences in the educational level between older women who practice a high level of physical activity and those who carry out a moderate or low level.
(b) If there are differences in the economic resources available between older women who practice a high level of physical activity and those who do a moderate or low level.
(c) If there are differences in the benefit of social and health services between older women who practice a high level of physical activity and those who do a moderate or low level.

## 2. Method

### 2.1. Participants

The cross-sectional study was carried out with the participation of 257 older women in the region of Alicante (Spain) with a mean age of 69.44 years (SD = 4.61), the minimum age being 61 years and the maximum age 93 years. Regarding their marital status, 56.4% of them were married, 23.3% widows, 8.2% single, 7.8% divorced, and 4.3% had another situation. There were 41.2% of the participants who lived alone.

Three inclusion criteria were established for the selection of participants: (a) to be 60 years old or above 60; (b) to be physically active; and (c) to have been practicing physical activity for more than one year. The exclusion criteria were: (a) anyone under 60 years of age; (b) older adults who showed that they were verbally inactive or sedentary as well as those who did not participate in physical activity; and (c) older adults with no ability to read the battery of questionnaires that needed to be answered.

In relation to their health status, the incidence of unhealthy lifestyle habits (tobacco and alcohol) and their clinical status (physical and psychological illnesses) were analyzed. Regarding their habits, 93% of the participants were non-smokers and only 4.3% smoked more than five cigarettes a day; in addition, 59.5% of them never consumed alcohol, although 19.1% drank it daily. Regarding their clinical situation, 70.4% did not suffer from any physical illness and 85.6% lacked psychological problems; specifically, the physical ailments with the highest incidence were osteoarthritis (9.7%), osteoporosis (4.3%) and hypertension (3.1%), and mental ailments depression (10.5%) and anxiety (3.5%).

### 2.2. Instruments

#### 2.2.1. International Physical Activity Questionnaire, IPAQ

The Physical Activity Questionnaire [24] assesses three types of physical activity: low intensity (like walking), moderate intensity, and high intensity. Participants can be classified into three exercise levels: high, moderate, and low. These different intensities of activities are regarded as follows: <3 METs, 3–6 METs, and >6 METs, respectively [25]. Women who practiced at least one more hour of moderate intensity activity daily above the baseline activity level, or half an hour of a high intensity activity above the baseline daily levels were classified in the group of high exercise levels. Regarding moderate activity levels were those who practiced at least half an hour of physical activity of moderate intensity almost every day.

Finally, those with low activity levels were considered as those who did not practice moderate or high activity levels [26]. The brief version of this questionnaire that used for this research asked participants to answer seven items related to the physical activity that they had practiced during the past seven days such as the question “how many days did you walk for at least 10 min in a row?” The brief version of the IPAQ has a reliability coefficient of 0.65 (rs = 0.76; 95% CI: 0.73–0.77). This questionnaire has also been used in recent studies on older adults [27].

#### 2.2.2. Brief Questionnaire on Quality of Life, CUBRECAVI

This questionnaire covers 21 different subscales assembled into nine scales among which subjective health and functional skills (functional autonomy and activities of daily living) were considered for the performance of this study. This questionnaire is highly recommended to evaluate the quality of life [28]. A member of the study must estimate the degree of satisfaction or the rate of the different matters that are considered. Participants must value the degree of satisfaction or the frequency of the items. We found questions such as “mark your household income” on a Likert response scale from 0 “nothing” to 3 “a lot of”. The extent of the questionnaire was approximately 20 min. The levels of internal consistency of the scales ranged between 0.70 and 0.92. This questionnaire was used recently to assess the quality of life in older adults [29].

### 2.3. Procedure

From 38 sports and cultural facilities in Alicante that were asked for their participation in this cross sectional study, only 18 agreed to collaborate. After testing that all the inclusion criteria were accomplished, they were given an informed consent form and questionnaire that they had to complete individually. They were also provided with a description of the ongoing research and its objectives. The study was conducted according to the guidelines of the Declaration of Helsinki and was approved by the Institutional Review Board (or Ethics Committee) of Miguel Hernández University 200115191342.

The members of the study had to answer and return the questionnaires after having practiced physical activities. The questionnaires were returned in an envelope in a follow-up appointment to maintain the privacy of the information.

### 2.4. Data Analysis

To assess the disparities among the members of this study according to their level of physical activity, the chi-square test was applied. To estimate the effect size, the Phi Coefficient and Cramer’s V (depending on the size of the contingency tables) were taken.

The established significance value was <0.05.

The data analyses were performed with the SPSS statistical package, version 23.0. (IBM corp., Armonk, NY, USA for Windows).

## 3. Results

### 3.1. Education Level

The examination of the scores that evaluated the educational level of the participants (Table 1) showed that there were statistically significant differences in the studies and education they had received and the level of physical activity they practiced (χ^2^ (8, *n* = 257) = 37.09; *p* < 0.001) with a small association between variables (VCramer = 0.269). It was examined in which educational degrees or level they differed according to their activity and the results indicated that this occurred between the degree of those who did not have primary studies and the other degrees (χ^2^ (2, *n* = 257) = 28.41; *p* < 0.001) with an association between the median variables (VCramer = 0.332) being the differences between those of high activity with those of moderate/low (20.2% vs. 52.3%) (χ^2^ (1, *n* = 257) = 26.74; *p* < 0.026; Phi = 0.323) and between those of moderate activity with those of high/low (55.2% vs. 26.2%) (χ^2^ (1, *n* = 257) = 22.33; *p* < 0.001; Phi = −0.295); and between the degree of those who attended high school with the other degrees (χ^2^ (2, *n* = 257) = 9.20; *p* = 0.010) with a small association between the variables (VCramer = 0.189) being the differences between those with high activity with those of moderate/low (14.4% vs. 3.9%) (χ^2^ (1, *n* = 257) = 9.10; *p* = 0.003; Phi = 0.188) and among those of moderate activity with those of high/low (4.3 % vs. 11.3%) (χ^2^ (1, *n* = 257) = 4.20; *p* = 0.040; Phi = −0.128); and among those who attended intermediate or university degrees with the other degrees (χ^2^ (2, *n* = 257) = 10.81; *p* = 0.004) with a small association between the variables (VCramer = 0.205) being the differences between high activity with those of moderate/low (28.9% vs. 13.7%) (χ^2^ (1, *n* = 257) = 8.90; *p* = 0.003; Phi = 0.186); and among those of moderate activity with those of high/low (11.2% vs. 27.0%) (χ^2^ (1, *n* = 257) = 9.92; *p* = 0.002; Phi = −0.196).

### 3.2. Income

In the scale relative to the economic resources available to the participants (Table 2), the research has shown that there were statistically significant differences in the monthly income available according to the level of physical activity they practiced (χ^2^ (4, *n* = 252) = 15.65; *p* = 0.004) with a small association between the variables (VCramer = 0.176). We examined which income ranges differed according to their activity and the results indicate that they occurred between the range of which their income did not exceed €600 with the other ranges (χ^2^ (2, *n* = 252) = 11.36; *p* = 0.003) with a small association between the variables (VCramer = 0.212) being the differences between those of high activity with those of moderate/low (12.9% vs. 27.8%) (χ^2^ (1, *n* = 252) = 7.92; *p* = 0.005; Phi = −0.177) and between those of moderate activity with those of high/low (31.3% vs. 13.9%) (χ^2^ (1, *n* = 252) = 11.14; *p* = 0.001; Phi = 0.210); and between the range of those whose income exceeded €1200 with the other ranges (χ^2^ (2, *n* = 252) = 10.69; *p* = 0.005) with a small association between the variables (VCramer = 0.206) being the differences between those of high activity with those of moderate/low (48.5% vs. 33.8%) (χ^2^ (1, *n* = 252) = 5.49; *p* = 0.019; Phi = 0.148) and between those of moderate activity with those of high/low (28.7% vs. 48.9%) (χ^2^ (1, *n* = 252) = 10.67; *p* = 0.001; Phi = −0.206).

### 3.3. Social and Health Services

In the subscale that evaluated the frequency with which the participants used social and health services (Table 3), the analyses showed that there were no statistically significant differences in their use according to the level of physical activity they practiced (χ^2^ (4, *n* = 257) = 7.34; *p* = 0.019; VCramer = 0.119) (Table 3).

Regarding the assessment of satisfaction with the use of services (Table 4), given the small number of participants who said that they were not satisfied at all with the three levels of activity, that degree of satisfaction was clustered with those who were somewhat satisfied, the variable being recoded into three categories: not at all/somewhat satisfied, quite satisfied, and very satisfied. The analyses showed that there were statistically significant differences in the satisfaction that the participants had with these services according to their level of physical activity (χ^2^ (4, *n* = 257) = 14.82; *p* = 0.005) with a small association between the variables (VCramer = 0.170). The analysis to understand to what degrees of autonomy were these differences in activity indicated that they were registered between the degree of those who were not at all/somewhat satisfied with the other degrees (χ^2^ (2, *n* = 257) = 9.30; *p* = 0.010) with a small association between the variables (VCramer = 0.190) being the differences between those with low activity and those with high/moderate (46% vs. 22.3%) (χ^2^ (1, *n* = 257) = 9.30; *p* = 0.002; Phi = 0.190); and between the degree of those who were quite satisfied with the other degrees (χ^2^ (2, *n* = 257) = 7.60; *p* = 0.022) with a small association between the variables (VCramer = 0.172) being the differences between those of high activity with those of moderate/low (62.5% vs. 47.1%) (χ^2^ (1, *n* = 257) = 5.93; *p* = 0.015; Phi = 0.152) and between those of low activity with those of high/moderate (37.8% vs. 55.9%) (χ^2^ (1, *n* = 257) = 4.16; *p* = 0.041; Phi = −0.127).

## 4. Discussion

The main goal of this study was to evaluate whether women who practiced a high level of physical exercise was related to high levels of quality-of-life socio-environmental factors.

First, the results showed that women with high levels of education practiced more physical activity. These findings are in line with other studies indicating that the level of education is related to the practice of physical exercise [12,13]. Even so, it should be noted that there is little research in this field. One study carried out with young women aged from 25 to 65 years old found similar results: the higher the educational level, the greater the practice of physical activity [16]. In this regard, various authors have emphasized that there are other factors, in addition to academic ones, that can motivate or stop older people from exercising such as lack of time, fatigue, financial restrictions, low motivation, or lack of facilities [30].

Second, the relationship between income and the level of physical activity was investigated. This study indicates that high income in older women is associated with high levels of physical activity. Similarly, participation of less than 600 MET-minutes was reported in Brazilian women due to their little physical activity in their free time [20]. The same results were found in a meta-analysis performed from 19 investigations [18] and in a study that associated low income with sedentary lifestyle in 1501 Korean older adults [18]. Like numerous investigations [30], our study suggests that the level of income in older women is a factor that can predict a higher practice of physical exercise.

Third, our data regarding satisfaction with social and health services showed that women with high levels of physical activity were more satisfied with themselves than those with low levels of physical activity. On this occasion, there was also a lack of studies on older adult women practicing physical activity in relation to the influence of social health services.

Regarding the effects that physical activity has on the health of older adults, it should be noted that it promotes functional abilities and autonomy [31], mitigates the effects of certain chronic diseases [32], improves physical, mental and general health [33], and increases the practice of healthy leisure activities [34].

The present investigation, however, has several limitations. One of these is that it is a cross-sectional study, which limits the possible extrapolations as the sample consisted of older women who practiced physical activity, does not represent the rest of older women; furthermore, the age range of the women in the sample is quite wide.

Despite the limitations of the study, it is worth highlighting the lack of similar studies, together with the need to implement them since, as has been said, it is a population especially sensitive to dependency, low self-esteem, etc. Therefore, it can be deduced that the promotion of research on the social health issues that surround older women, whether or not they practice physical activity regularly, can help to improve existing programs, generate new approaches, and facilitate adherence to them.

For future research, the use of biometric methods should be considered to evaluate the practice of physical activity (step counting, triaxial accelerometers, etc.) as well as to expand the evaluation of measures that lead to the above-mentioned inequality (family conciliation, care of the house or dependent grandchildren, among others). Therefore, a future line of research is the analysis of the factors that encourage older adult women not to practice physical activity and relegate their health care as well as whether these issues are related to multitasking or other facts of daily life.

However, our study points out the need to continue research along this line to prove the necessity for physical activity policies to consider both the barriers and the socio-sanitary facilitators that prevent or facilitate the practice of physical activity in women. These policies should be directed at minimizing the negative impact that the low economic status and the low level of education have on the practice of physical exercise. Consequently, special attention should be paid to disadvantaged population groups.

## 5. Conclusions

This research shows that older women with high levels of physical activity are related to higher quality of life socio-environmental factors, since they register high levels of education and income. Likewise, it has been shown that the level of physical exercise they perform is associated with satisfaction with social and health services. In the same way, the study showed more dissatisfaction with the use of social and health services associated with low activity levels. All this suggests that this study is unique in the subject it addresses, since it shows the profile of social inequalities in older women as well as their better or worse quality of life, depending on the level of physical activity they practice. Another of the conclusions that can be drawn from the present work is the need to continue investigating the relationship between certain social factors (low level of income and education) with the practice (or not) of physical exercise in older women and the implications that this fact has on its health.

## Figures and Tables

**Table 1:** Frequencies of the education scale (CUBRECAVI) according to their level of physical activity.

Education Scale	No Primary Studies		Primary Studies		Vocational Training		Elementary and Higher Baccalaureate (A Levels)		University Degree Studies	
	*n*	%	*n*	%	*n*	%	*n*	%	*n*	%
High Ph.A	21	20.2	26	25.0	12	11.5	15	14.4	30	28.9
Moderate Ph.A	64	55.2	28	24.1	6	5.2	5	4.3	13	11.2
Low Ph.A	16	43.3	9	24.3	3	8.1	1	2.7	8	21.6

*n* = number of participants, % = percentage, Ph.A = Physical Activity.

**Table 2:** Frequencies of the income scale (CUBRECAVI) according to their level of physical activity.

Income Scale	Up to €600		Between €601 and €1200		More than €1200	
	*n*	%	*n*	%	*n*	%
High Ph.A	13	12.9	39	38.6	49	48.5
ModeratePh.A	36	31.3	46	40.0	33	28.7
Low Ph.A	6	16.7	12	33.3	18	50.0

*n* = number of participants, % = percentage.

**Table 3:** Frequencies of the service use subscale (CUBRECAVI) according to their level of physical activity.

Service Use Subscale	Never		Occasionally Frequently		Occasionally Frequently	
	*n*	%	*n*	%	*n*	%
High Ph.A	5	4.8	62	59.6	37	35.6
ModeratePh.A	2	1.7	62	53.5	52	44.8
Low Ph.A	4	10.8	20	54.1	13	35.1

*n* = number of participants, % = percentage.

**Table 4:** Frequencies of the subscale of satisfaction with the use of services (CUBRECAVI) according to their level of physical activity.

Satisfaction	No/Somewhat Satisfied		Quite Satisfied		Very Satisfied	
	*n*	%	*n*	%	*n*	%
High Ph.A	23	22.1	65	62.5	16	15.4
ModeratePh.A	26	22.4	58	50.0	32	27.6
Low Ph.A	17	46.0	14	37.8	6	16.2

*n* = number of participants, % = percentage.

## Data Availability

The database is under the custody of the IP with anonymized data.
